# Diagnostic and Therapeutic Value of the Exercise-Induced Myokine Irisin in Cancer Biology: A Comprehensive Review

**DOI:** 10.3390/diseases13090304

**Published:** 2025-09-16

**Authors:** Wesam F. Farrash, Ahmad A. Obaid

**Affiliations:** 1 Department of Clinical Laboratory Sciences, Faculty of Applied Medical Sciences, Umm Al-Qura University, Holy Makkah P.O. Box 7607, Saudi Arabia; 2 Department of Physiology, Faculty of Medicine, Umm Al-Qura University, Holy Makkah P.O. Box 7607, Saudi Arabia; aaobaid@uqu.edu.sa

**Keywords:** myokines, Fndc5, exercise, Warburg effect, PI3K/Akt/mTOR pathway

## Abstract

**Objectives:** Cancer is a multifactorial disease determined by several factors. Metabolic disorders such as obesity and diabetes significantly contribute to cancer risk by promoting chronic inflammation, insulin resistance, and hormonal dysregulation. Obesity and hyperglycaemia elevate insulin-like growth factor-1 (IGF-1) levels, driving oncogenic pathways such as PI3K/Akt/mTOR, which promote tumour proliferation and survival. Furthermore, cancer cells undergo metabolic reprogramming, characterised by increased reliance on glycolysis (Warburg effect), facilitating tumour growth and therapy resistance. Hence, body weight reduction and glycaemic control may represent potential strategies for cancer prevention and treatment. Irisin, a myokine secreted by skeletal muscle, plays a critical role in cellular metabolism and energy homeostasis. Emerging evidence suggests that irisin may exert tumour-suppressive effects by modulating key metabolic and oncogenic pathways. **Methods:** A systematic literature search identified studies investigating irisin’s effects in various cancer models. **Results:** In vitro, irisin exerts dose- and time-dependent anti-proliferative effects in a variety of cancer cell lines, primarily via PI3K/Akt/mTOR inhibition and AMPK activation, leading to cell cycle arrest and apoptosis. Additionally, irisin inhibits epithelial–mesenchymal transition, which suppresses cancer cell migration and invasion. However, conflicting findings, particularly in hepatocellular carcinoma, suggest tissue-specific responses. Similarly, clinical data regarding systemic and tumoural irisin levels remain inconsistent and appear to vary based on cancer type and stage. **Conclusions:** Irisin represents a promising therapeutic target due to its ability to modulate metabolic and oncogenic pathways. However, further research is needed to elucidate its clinical relevance and optimise its application as an adjunct to existing cancer therapies.

## 1. Introduction

Cancer is a multifactorial disease and a major public health concern worldwide, resulting from the interplay of genetic mutations and environmental factors [1,2,3]. Metabolic disorders, particularly obesity and diabetes, have been strongly linked to increased cancer risk due to their impact on systemic inflammation, insulin resistance, and hormonal dysregulation [3,4,5]. Obesity-induced hyperinsulinemia elevates insulin-like growth factor-1 (IGF-1) levels, which in turn promotes tumour growth and progression [4,5]. Additionally, obesity and type 2 diabetes contribute to a pro-inflammatory tumour microenvironment through elevated cytokine levels and adipokine dysregulation, further driving oncogenesis [3,4,5].

Several metabolic factors play pivotal roles in obesity-associated carcinogenesis [6,7,8,9,10]. Hyperinsulinemia and hyperglycaemia can enhance tumour cell proliferation by stimulating the phosphatidylinositol-3-kinase (PI3K) signalling cascade that stimulates the mammalian target of rapamycin (mTOR) through the protein kinase B (Akt) [4,5]. This oncogenic pathway regulates cell growth, survival, and metabolism [6,7,8,9,10]. Chronic activation of this pathway in metabolic disorders promotes cancer cell proliferation and inhibits apoptosis. IGF-1, a growth factor, further amplifies these effects by activating mitogenic and anti-apoptotic signalling cascades [6,7,8,9,10]. The Warburg effect is another metabolic alteration that is observed in cancer, wherein cells mainly depend on glycolysis for energy production even in oxygen-rich conditions. These metabolic alterations enhance tumour growth and resistance. Therefore, targeting these metabolic pathways presents a promising avenue for cancer treatment [11,12,13].

In response to physical activity, skeletal muscle releases a class of bioactive molecules termed myokines, which are integral to mediating the systemic benefits of exercise [14,15]. Among these, irisin has garnered considerable interest due to its involvement in energy regulation and its putative anticancer properties. Irisin is a 112-amino-acid peptide cleaved from the fibronectin type III domain-containing protein 5 (Fndc5), a transmembrane protein predominantly expressed in skeletal muscle, but also detectable in the adipose tissue, heart, and brain [14]. Emerging evidence has revealed irisin expression in additional organs including the pancreas, liver, stomach, spleen, ovaries, testes, skin, and retina. However, its levels in these tissues are markedly lower than those observed in skeletal muscle [16]. Notably, adipose tissue expresses irisin at levels more than 100-fold lower than skeletal muscle [17,18].

Irisin primarily exerts its biological functions by binding to cell surface integrins, a family of receptors widely expressed across different tissues. The integrin αVβ5 complex has been most consistently identified as the principal receptor mediating irisin’s effects in various cell types, including adipocytes, osteocytes, osteoclasts, and astrocytes [19,20]. Additionally, irisin is proposed to interact with αVβ1 and α5β1 integrins, especially in mesenchymal-derived tissues such as skeletal muscle, bone, and adipose tissue, where it contributes to metabolic remodelling [19,20]. Binding of irisin to αVβ5 may occur through a two-step mechanism involving extracellular HSP90α, a molecular chaperone released from skeletal muscle during exercise that facilitates and stabilizes the receptor–ligand interaction [19,20].

**Figure 1:**
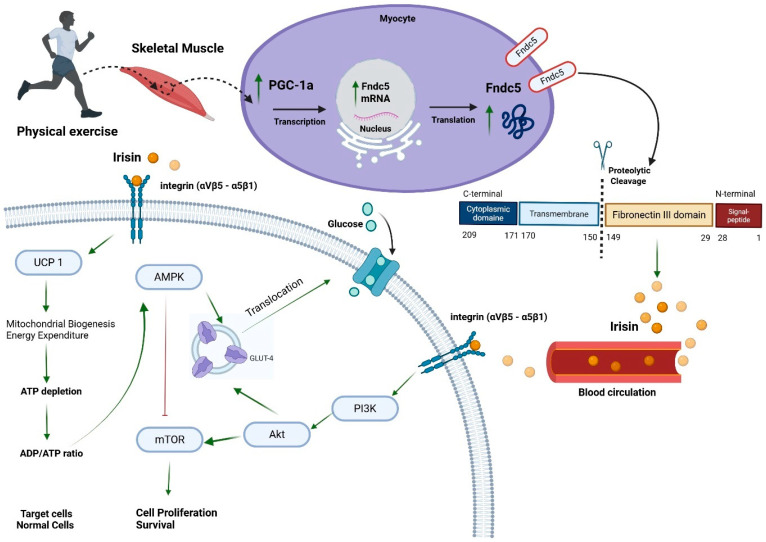
Irisin secretion and signalling pathway. Peroxisome proliferative-activated receptor gamma coactivator 1-alpha (PGC-1a), fibronectin type III domain-containing protein 5 (Fndc5), uncoupling protein 1 (UCP 1), AMP-activated protein kinase (AMPK), mammalian target of rapamycin (mTOR), phosphatidylinositol-3-kinase (PI3K), protein kinase B (Akt), and glucose transporter 4 (GLUT-4). ↑ green arrow means induction. ┴ red line means inhibition. Created in BioRender. Farrash, W. (2025) https://BioRender.com/srowu50.

Given that skeletal muscle is the primary source of irisin, significant inter-organ crosstalk has been observed. One prominent example is irisin’s ability to promote browning of white adipose tissue (WAT), characterized by increased uncoupling protein-1 (UCP1) expression and mitochondrial biogenesis, leading to elevated energy expenditure [14,15,21]. Beyond adipose tissue, irisin mediates metabolic effects through the PI3K/Akt/mTOR signalling cascade and its counter-regulator AMP-activated protein kinase (AMPK). In the liver, irisin inhibits gluconeogenesis while enhancing glycogen synthesis and fatty acid oxidation, thereby improving insulin sensitivity and reducing hepatic lipid accumulation [16] (Figure 1).

In the pancreas, irisin facilitates β-cell proliferation, enhances insulin secretion and survival, and exhibits anti-inflammatory and antioxidant properties, thereby contributing to overall pancreatic health [19]. Moreover, irisin has demonstrated broader anti-inflammatory effects, notably through the downregulation of pro-inflammatory cytokines, including interleukin-6 and interleukin-1β, across multiple tissues [20,22,23]. Evidence from both clinical and experimental studies further underscores irisin’s role in bone metabolism, where it promotes osteoblast differentiation, enhances bone mineral density, reduces osteocyte apoptosis, and stimulates bone remodelling via upregulation of key osteogenic transcriptional factors [20,22,23].

Given that cancer cells preferentially rely on glycolysis for energy production, irisin-induced metabolic shifts towards oxidative phosphorylation may have tumour-suppressive effects [11,12,13]. Additionally, irisin has been found to modulate the PI3K/Akt/mTOR pathway, a critical signalling cascade that regulates cell growth, survival, and metabolism, thereby inhibiting oncogenic processes [24,25,26]. Furthermore, irisin has been reported to influence IGF-1 signalling, which plays a critical role in cancer development by promoting mitogenic and anti-apoptotic pathways [27,28]. While its precise role in carcinogenesis remains under investigation, current evidence suggests that irisin may exert direct and indirect anticancer effects, making it a promising target for cancer prevention and therapy.

## 2. Search Strategy

Relevant studies on irisin and cancer were sourced by searching the electronic data databases ‘**PubMed**’, ‘**Scopus**’, and ‘**Web of Science**’. The search terms included ‘*irisin*’ or ‘*Fndc5*’ with ‘*cancer*’, ‘*tumour*’, ‘*neoplasia*’, ‘*oncogenesis*’, ‘*cell proliferation*’, ‘*apoptosis*’, ‘*metastasis*’, ‘*epithelial-mesenchymal transition (EMT)*’, ‘*IGF-1*’, and ‘*PI3K/Akt/mTOR pathway*’. These terms were used in various combinations to retrieve studies published from 2012 to the present. The reference lists of all chosen publications were further examined to identify further studies relevant to the role of irisin in cancer.

## 3. Structure, Source, Half-Life, and Biological Actions of Irisin

Irisin is a myokine produced by the cleavage of Fndc5, a transmembrane protein predominantly expressed in skeletal muscle cells [29,30]. Upon physical exercise, Fndc5 undergoes proteolytic processing, releasing irisin into the bloodstream [29,30]. Structurally, irisin comprises 112 amino acids, forming a crucial domain for its biological activity, fibronectin type III. Notably, irisin undergoes N-linked glycosylation, a post-translational modification essential for its stability and function [29,30] (Figure 1).

Since skeletal muscle is the primary source of irisin, the secretion of irisin is markedly increased during physical activity, linking muscle contraction to various systemic effects [20,31,32]. Despite its identification over a decade ago, the exact half-life of irisin in human circulation remains under investigation, with estimates suggesting a relatively short duration, necessitating continuous production for sustained physiological effects [20,31,32].

Irisin plays a pivotal role in energy metabolism, particularly concerning obesity and insulin resistance [20,21,32]. One of its primary actions is promoting the browning of white adipose tissue, which enhances heat production and energy expenditure, thereby reducing obesity [20,21,32]. Moreover, irisin enhances glucose uptake in skeletal muscle by upregulating glucose transporter type 4 expression, improving insulin sensitivity. This action is particularly beneficial in mitigating insulin resistance, a precursor to type 2 diabetes mellitus [20,21,32].

## 4. Obesity-Induced Carcinogenesis

Obesity is an established risk factor for multiple solid tumours [3]. One of the key mechanisms linking obesity to cancer is the altered secretion of adipokines, bioactive molecules produced by adipose tissue, which play pivotal roles in tumour initiation and progression [33,34,35,36]. Among these, adiponectin, an anti-inflammatory adipokine, has demonstrated antitumour effects by activating AMPKα, thereby suppressing metabolic activity and inhibiting cellular proliferation in several malignancies [37,38,39]. Conversely, leptin and resistin, which are pro-inflammatory adipokines, contribute to tumour-promoting processes by activating the PI3K/Akt/mTOR signalling pathway, thereby sustaining tumour cell survival, growth, and invasion [33,34,35,36]. These findings support the notion that weight reduction and metabolic regulation may serve as effective strategies to prevent or slow the progression of obesity-associated neoplasia [3,7,10]. In addition to its well-documented role in adipose tissue regulation, irisin has also been identified as an adipokine with potential involvement in obesity-related tumourigenesis [40,41]. The subsequent sections will explore in detail the molecular and clinical evidence linking irisin expression to cancer development and progression across various tissue types.

## 5. In Vitro Effects of Irisin on Cancer Cells

Since its discovery, recombinant irisin has been utilized in various cancer cell lines to assess its anticancer potential. Research has primarily focused on its anti-proliferative effects, with additional investigations exploring its impact on invasion, migration, metastasis, and cell cycle regulation.

### 5.1. Irisin’s Role in Proliferation

Despite inconsistencies in irisin levels reported in humans and animal models following physical activity, its physiological concentration is estimated to be 3–5 ng/mL. Cancer cell lines have been exposed to both physiological and supraphysiological concentrations of irisin to evaluate its role as an anticancer agent. Interestingly, supraphysiological irisin concentrations were generally more effective in most studies. However, even low concentrations (2.5 nM) significantly reduced breast cancer cell viability (MDA-MB-231 and MCF-7) after 24 h, as shown in ref. [42], detailed below (Table 1).

In prostate cancer cells, Tekin et al. examined the effects of irisin (0.1–100 nM) on androgen receptor-positive (LNCaP) and -negative (DU-145 and PC3) cancer cell lines, reporting a significant reduction in cell viability at supraphysiological concentrations (10 nM and 100 nM) after 24 h treatment [43]. Treating metastatic PC3 prostate cancer cells with variable irisin concentrations (5 to 100 nmol/L) for 24, 48, and 72 h in another study also promoted cell death and upregulated several markers of apoptosis in vitro and in a xenograft animal model [44]. However, others reported that viability of LNCaP and DU-145 prostate cancer cells was reduced significantly following treatment with low irisin concentrations (5 and 10 nM) [45].

In pancreatic cancer cells, 12 h treatment with 100–200 nM irisin decreased cell viability in the PANC-1 cell line [46]. Liu et al. also treated MIA PaCa-2 and Panc03.27 pancreatic cancer cells with 0–100 nM of glycosylated and non-glycosylated irisin isoforms for 24, 48, and 72 h, observing a dose- and time-dependent reduction in MIA PaCa-2 viability, while Panc03.27 exhibited only a time-dependent effect [47]. However, 50 nM of irisin was enough to reduce cell viability and increase apoptotic markers on PANC-1 and BxPC-3 after 48 h treatment [24] (Table 1).

Although ovarian cancer cell lines (OVCAR3, SKOV3, and Caov4) showed no significant changes in viability at 24 h, 48 to 120 h treatments induced a significant dose- and time-dependent reduction, with the minimum effective dose reported as a 15 nM concentration [48]. In another study, A2780 ovarian cancer cells likewise exhibited reduced cell numbers at both physiological (5 and 10 nM) and supraphysiological concentrations (12.5–100 nM), while SKOV3 cells only responded to supraphysiological doses (50–100 nM) after 48 h [26].

Glioblastoma cell lines (U-87 MG, T98G, and LN-18) displayed a dose-dependent reduction in viability after being exposed to 200–1000 nM irisin for 72 h, with U-87 MG cells showing the highest sensitivity at 1000 nM concentration [49]. In osteosarcoma cell lines (U2O2 and MG-63), 12 h treatments had no significant effect, whereas 48 h treatments at 50 and 100 ng/mL significantly reduced cell viability [50]. Moreover, 200 ng/mL irisin exerted a stronger inhibitory effect at both 24 and 48 h, findings corroborated by Cheng et al., who confirmed the anti-proliferative effects of irisin in osteosarcoma cells [51]. Lung cancer cell lines, including non-small-cell lung cancer (NSCLC) models, also demonstrated reduced cell viability following irisin treatment [52,53] (Table 1).

### 5.2. Molecular Mechanisms of Irisin-Induced Growth Inhibition

Mechanistically, cancer cell proliferation is largely driven by dysregulated activation of the PI3K/Akt/mTOR signalling pathway, which regulates cell metabolism, growth, survival, and motility. Irisin has been shown to inhibit PI3K/Akt phosphorylation in ovarian [26], pancreatic [24], and lung [52] cancer cell lines. Additionally, Liu et al. reported that irisin activated AMPK, leading to mTOR inhibition in pancreatic cancer cells [47]. Irisin has also been shown to induce G0/G1 phase arrest in pancreatic cancer cells, contributing to proliferation inhibition, which correlated with reduced cyclin D1 expression [47]. In glioblastoma cells, 48 h treatments led to G2/M phase arrest, accompanied by a significant increase in p21 gene expression [49] (Table 1).

Despite these promising findings, conflicting reports exist. Moon et al. tested irisin on obesity-related cancers (colorectal, thyroid, and oesophageal cancer) and observed no effect on proliferation [54]. Moreover, Shi et al. reported that HepG2 hepatocellular carcinoma cells exhibited increased proliferation upon irisin treatment, attributed to PI3K/Akt pathway activation, highlighting potential tissue-specific responses [55] (Table 1).

### 5.3. Irisin and Cancer Cell Invasion and Metastasis

Cancer cells metastasize through EMT, a process characterized by E-cadherin downregulation and N-cadherin/vimentin upregulation, regulated via STAT3/Snail activation [56,57]. Irisin treatment suppressed EMT, invasion, and metastasis in MIA PaCa-2 and Panc03.27 pancreatic cancer cells, leading to reduced migration in scratch wound assays and the trans-well invasion assay [47]. Similarly, ovarian cancer cells demonstrated decreased metastatic potential, with irisin inhibiting PI3K/Akt signalling and downregulating metalloproteinase (MMP)2 and MMP9 [26,48].

In osteosarcoma, irisin reversed interleukin-6 (IL-6)-induced EMT, leading to a reduction in N-cadherin, vimentin, fibronectin, MMP2, MMP6, and MMP9 expression, alongside STAT3/Snail suppression [50]. Similar effects were observed in lung cancer cells, where PI3K/Akt/Snail inhibition diminished EMT markers [52] (Table 1). However, contradictory results have been reported. In HepG2 hepatocellular carcinoma cells, irisin enhanced invasion and metastasis via PI3K/Akt upregulation, further reinforcing the notion of tumour-specific effects [55].

## 6. Systemic Irisin Levels and Its Expression in Clinical Cancer Specimens

Accumulating evidence suggests that serum irisin levels and Fndc5/irisin expression exhibit significant alterations in various malignancies, with notable reductions observed in multiple cancer types (Table 2 and Table 3). In breast cancer, the serum from 101 patients diagnosed with invasive ductal carcinoma exhibited significantly lower irisin levels compared to samples collected from 51 healthy women [58]. Similar findings were reported in a cohort of 148 breast cancer patients with spinal metastases, reinforcing the hypothesis that a low serum level of irisin may be associated with tumour progression and metastatic potential [59]. At the tissue level, however, irisin protein expression was elevated in archived breast, cervical, endometrial, and ovarian cancer tissue specimens (*n* = 10/cancer type) [60]. In colorectal cancer (CRC), while a study involving 76 patients, particularly obese individuals, also demonstrated markedly reduced serum irisin levels relative to 40 healthy controls [61], another report revealed increased irisin protein expression in 222 CRC specimens [62], suggesting an inverse relationship between irisin serum levels and tissue expression. Likewise, Esawy et al. and Aslan et al. observed significant declines in serum irisin levels among 150 bladder cancer [63] and 80 prostate cancer patients [64], respectively, further supporting a potential tumour-suppressive role for irisin (Table 2 and Table 3).

Conversely, findings in hepatocellular carcinoma (HCC) remain inconsistent. While Gaggini et al. and Shi et al. found no significant difference in serum irisin levels between 56 HCC patients and healthy controls [55,65], studies involving 262 patients reported substantially lower circulating irisin levels [66,67], suggesting a potential link between irisin suppression and disease severity (Table 3). However, Gaggini et al. and Shi et al. reported upregulated levels of Fndc5 gene expression in HCC [55,65], while the extracted data indicated low levels of Fndc5 gene expression in HCC [66].

Contrastingly, Shahidi et al. noted an increase in serum irisin levels in 51 newly diagnosed gastric cancer patients [68], implying that irisin upregulation may contribute to early tumourigenesis. Aydin et al.’s findings confirm the relation, since irisin was highly expressed in gastrointestinal cancer tissues such as oesophageal, pancreatic, and grade II astrocytoma tissues compared to normal controls, suggesting a context-dependent role in cancer progression [69]. Similarly, Altay et al. found increased serum irisin levels in 176 renal cancer patients, indicating a possible role in renal cancer metabolism [70]. However, the protein expression of irisin was reduced in renal cancer tissue in the only study to report this pattern [71]. Moreover, heightened Fndc5/irisin expression was observed in thyroid [72] and lung [73] tissues compared to normal controls, suggesting a context-dependent role in cancer progression (Table 2 and Table 3).

**Table 2:** List of studies that measured irisin expression in clinical cancer specimens. Immunohistochemistry (IHC). ↑ = significant increase; ↓ = significant decrease.

Tumour Tissue	Fndc5/Irisin	Main Results	References
Breast, Cervix, Ovaries, Endometrium	↑ irisin expression IHC	Breast and reproductive tract cancer	[60]
Colorectal Cancer	↑ irisin expression IHC	CRC compared to normal tissue	[62]
Oesophagus, Stomach, Liver, Pancreas, Brain	↑ irisin expression IHC	Gastrointestinal cancer, grade II astrocytoma	[69]
Thyroid Cancer	↑ irisin expression IHC	In oncolytic papillary carcinoma, anaplastic carcinoma	[72]
Lung Cancer	↑ Fndc5 mRNA	In malignant tissue compared to non-malignant Higher in AC in comparison to SSC	[73]
Hepatocellular Carcinoma	↑ Fndc5 mRNA	HCC patients compared to donors	[55]
	↑ Fndc5 mRNA	HCC patients compared to controls	[65]
	↓ Fndc5 mRNA	Extracted data from TCGA for HCC patients compared to controls	[66]
Renal Cancer	↓ irisin expression IHC	Chromophobe renal cell carcinoma	[71]

**Table 3:** List of clinical studies that measured serum irisin in cancer patients and their main findings. ↑ = significant increase; ↓ = significant decrease; and ↔ = no significant change.

Type of Cancer	Serum Irisin Levels	Number of Patients	Study Details	References
Breast cancer	↓	101	Patients with invasive ductal	[58]
	↓	148	Patients with spinal metastases	[59]
Colorectal cancer	↓	116	Obese and non-obese patients	[61]
Bladder cancer	↓	150	75 patients vs. 75 apparently healthy subjects	[63]
Prostate cancer	↓	80	50 primary patients vs. 30 healthy male subjects	[64]
Hepatocellular carcinoma	↔	36	HCC patients vs. healthy control	[65]
	↔	20		[55]
	↓	219		[66]
	↓	43		[67]
Gastric cancer	↑	51	Newly diagnosed cases vs. healthy control	[68]
Renal cancer	↑	176	Different types of renal cancers vs. heathy samples	[70]

Collectively, these findings underscore that variations in irisin levels and expression may depend not only on the type and clinical stage of cancer but also on the methodological approaches employed across studies (Table 2 and Table 3). While biological variability is likely, a significant source of heterogeneity in reported outcomes appears to come from technical limitations related to irisin detection methods. Commonly used approaches, including the enzyme-linked immune assay (ELISA), immunohistochemistry (IHC), and Western blotting (WB), are subject to inconsistent sensitivities and specificities depending on assay design, antibody selection, and sample processing protocols [74].

A particularly important factor is the nature of the primary antibody used and the targeted immunogen. Many early studies relied on polyclonal antibodies, which are prone to cross-reactivity due to their affinity for multiple epitopes, potentially detecting non-specific proteins and inflating measured irisin levels [75,76,77]. In contrast, most currently available commercial antibodies are mouse monoclonal IgG, offering improved specificity. Nonetheless, inconsistencies persist, in part due to mismatches between antibody epitope recognition and the experimental context in relation to the detection of cellular and/or secreted irisin protein [75,76,77].

In this context, it is crucial to align antibody choice with the detection method and targeted domain of irisin protein [76,77]. For body fluid assays such as ELISA, monoclonal antibodies targeting the irisin extracellular domain, specifically amino acid residues 30–140, are recommended for detecting secreted irisin with high specificity [76]. Conversely, tissue-based detection (e.g., IHC or WB in homogenized samples) should employ antibodies that recognize the intracellular domain (amino acids 150–209), particularly in studies involving membrane-bound or precursor forms of Fndc5/irisin [76]. Additionally, pre-analytical variables such as tissue fixation, antigen retrieval protocols, and storage conditions can further influence antigen detectability and signal quality in tissue-based study [78].

These methodological discrepancies may partially explain the conflicting reports regarding irisin’s diagnostic and prognostic significance in cancer. Therefore, standardized validation of detection protocols, especially through more specific methods (e.g., tandem mass spectrometry), is warranted to enable accurate measurement and comparison across the different sample types [74,75,76,77].

## 7. Conclusions

Although emerging evidence supports a role for irisin in cancer biology, the current body of literature remains limited, with inconsistent findings across malignancies. Elevated irisin protein expression has been reported in gastrointestinal, brain, breast, ovarian, cervical, endometrial, thyroid, and colorectal cancers, while renal cancer showed decreased levels. Gene expression data largely align with protein findings but remain sparse. Conflicting results, particularly in hepatocellular carcinoma, suggest that the role of irisin may be modulated by tumour-specific regulatory mechanisms.

However, these discrepancies may not solely reflect biological differences but are also likely influenced by methodological variability, including inconsistencies in tissue handling, timing of sample collection, and especially antibody specificity and assay design. Therefore, the standardization of detection protocols, including the use of validated monoclonal antibodies targeting specific epitopes and the incorporation of mass spectrometry-based confirmation methods, is essential to reduce technical bias and enhance reproducibility.

At the systemic level, while several studies report lower circulating irisin levels in cancer patients, others show elevated levels, further underscoring the need for cross-validation between serum, tissue, and gene expression data. To fully elucidate irisin’s diagnostic and prognostic value, future studies must integrate molecular data with clinicopathological features using harmonised methods.

In summary, while irisin shows promise as a diagnostic biomarker and a modulator of tumour progression, its clinical translation will require a concerted effort to standardise methodologies and conduct well-powered, longitudinal studies across diverse cancer types.

## 8. Future Directions

To advance irisin research from observational evidence to clinical application, the next phase of investigation must be built on methodological consistency and translational focus.

First, the detection protocols should be standardized. Future studies should adopt uniform, validated assays for irisin detection. This includes using monoclonal antibodies targeting well-defined epitopes (e.g., aa 30–140 for serum, aa 150–209 for tissues), and then using tandem mass spectrometry as an orthogonal method to confirm assay specificity and sensitivity. Altogether, detailed protocols for tissue fixation, antigen retrieval, and sample storage should be reported to enhance reproducibility.

Second, design large-scale, longitudinal studies. There is a critical need for prospective multicentre cohort studies that collect matched serum/tumour tissue samples, besides monitoring irisin dynamics over time in relation to treatment, recurrence, and survival. In addition, use consistent cut-offs for high vs. low irisin expression to enable pooled analyses.

Third, incorporate irisin into multi-analyte biomarker panels. This would enable assessment of additive value, evaluation of dose–response relationships between irisin levels and clinical outcomes, and exploration of interventional studies targeting irisin pathways through exercise, metabolic modulation, or pharmacological agents in cancers where irisin demonstrates functional relevance.

Finally, collaborative research networks are required. Cross-disciplinary collaboration between basic researchers, oncologists, pathologists, and biostatisticians is essential to develop consensus-driven protocols and facilitate translational trials.

Through these focused strategies, irisin may emerge as a reliable biomarker and therapeutic target in precision oncology.

## Figures and Tables

**Table 1:** List of in vitro studies and their outcome in various cancer cell lines. Bcl- associated X protein (BAX); B-cell lymphoma 2 (BCL2); metalloproteinase (MMP); Non-small lung cancer (NSCLCs); Nuclear factor-kappa B (NF-kB); light chain 3 (LC3 II); AMP-activated protein kinase (AMPK); mammalian target of rapamycin (mTOR); protein kinase P (Akt); hypoxia inducer factor-1 alpha (HIF-1α); signal transducer and activator of transcription 3 (STAT3); Phosphoinositide 3-kinase (PI3K); zinc finger transcription factor (Snail). ↓: significant decrease; ↑: significant increase; and NI: not investigated.

Type of Cancer	Cell Lines	Effective Concentration	Cell Viability	Cell Cycle	Apoptosis	EMT	Invasion	Migration	Signalling Pathway	Reference
Prostate	LNCaP DU-145 PC3	0.1–100 nM Effective: 10–100 nM	**↓**	**_NI**	**NI_**	**NI_**	**NI_**	**NI_**	**NI_**	[43]
	PC3	5–100 nM Effective: 100 nM	**↓**	**NI**	**↑**	**NI**	**NI**	**NI**	**↑** BAX **↑** Caspase-3 ↓ BCL2	[44]
	LNCaP DU-145	5–40 nM Effective: 5 and 10 nM	**↓**	**NI_**	**↑**	↓ MMP2 and 9	**NI_**	**NI_**	**NI_**	[45]
Pancreatic	Panc-1	0–200 nM Effective: 100–200 nM	**↓**	**NI**	Ferroptosis	**NI**	**NI**	**NI**	↓ NF-kB ↑ LC3 II	[46]
	PANC-1 BxPC-3	0–50 nM Effective: 50 nM	**↓**	**NI**	**↑**	**↓**	**↓**	**↓**	**↑** BAX ↓ BCL2 ↓ PI3K/Akt	[24]
	MIAPaCa-2 Panc03.27	0–100 nM Effective: 10 and 100 nM	**↓**	Arrest in G0/G1	**NI**	**↓**	**↓**	**↓**	**↑** AMPK ↓ mTOR	[47]
Ovarian	OVCAR3 SKOV3 Caov4	5–70 nM	**↓**	**NI**	↑ 10 nM on OVCAR3	↓ MMP2 and 9	**↓**	**NI**	↓ HIFI-α pathway	[48]
	A2780 SKOV3	5–100nM_	**↓**	**NI**	_**NI**	↓	**↓**	**↓**	↓ PI3K/Akt	[26]
Glioblastoma	U-87 MG T98G LN-18	200–1000 nM Effective: 1000 nM	**↓**	Arrest in G2/M	No effect	**NI**	**↓**	**NI**	↓ MMP-2 activity	[49]
Osteosarcoma	U2O2 MG-63	25–200 ng/mL Effective = 100 and 200 ng/ml	**↓**	**NI**	**NI**	**↓**	**↓**	**↓**	↑ p-STAT3/Snail	[50]
	U2OS	25–200 ng/ml	**↓**	**NI**	**NI**	**↓**	**↓**	**↓**	**NI**	[51]
Lung	A549	10–50 nM Effective = 20–50 nM	**↓**	**NI**	**NI**	**↓**	**↓**	**↓**	↓ PI3K/Akt/Snail	[52]
NSCLCs	A549 H358 H1299 H1650		**↓**	**NI**	**NI**	**NI**	**NI**	**NI**	↓ NF-kB	[53]
Breast	MDA-MB-231 MCF-7	0.62–20 nM Effective: 2.5–20 nM	**↓**	**NI**	**↑**	**↓**	**↓**	**↓**	↓ NF-kB ↑ Caspase-3/7 cleavage	[42]
Endometrial Colon Thyroid Oesophageal	KLE and RL95-2 HT29 and MCA38 SW579 OE13 and OE33 and BHP7	5–10 nmol/L 50–100 nmol/L	**NI**	**NI**	**NI**	**NI**	**NI**	**NI**	**NI**	[54]
Hepatocellular carcinoma	HepG2 SMCC7721	0.625–20 nM Effective: 20 nM	**↑**	**NI**	**↓**	**↑**	**↑**	**↑**	↑ PI3K/Akt	[55]

## References

[1] Bray F., Laversanne M., Weiderpass E., Soerjomataram I. (2021). The ever-increasing importance of cancer as a leading cause of premature death worldwide. Cancer.

[2] Bray F., Laversanne M., Sung H., Ferlay J., Siegel R.L., Soerjomataram I., Jemal A. (2024). Global cancer statistics 2022: GLOBOCAN estimates of incidence and mortality worldwide for 36 cancers in 185 countries. CA Cancer J. Clin..

[3] Ibrahim S.S., Ibrahim R.S., Arabi B., Brockmueller A., Shakibaei M., Büsselberg D. (2024). The effect of GLP-1R agonists on the medical triad of obesity, diabetes, and cancer. Cancer Metastasis Rev..

[4] Mishra R., Patel H., Alanazi S., Kilroy M.K., Garrett J.T. (2021). PI3K Inhibitors in Cancer: Clinical Implications and Adverse Effects. Int. J. Mol. Sci..

[5] Ashadul Sk M., Hemalatha K., Matada G.S.P., Pal R., Manjushree B.V., Mounika S., Haripriya E., Viji M.P., Anjan D. (2025). Current developments in PI3K-based anticancer agents: Designing strategies, biological activity, selectivity, structure-activity correlation, and docking insight. Bioorg. Chem..

[6] Kocianova E., Piatrikova V., Golias T. (2022). Revisiting the Warburg Effect with Focus on Lactate. Cancers.

[7] Saheed E.S., Aromolaran R.F., Atoyebi A.D., Adeleke F.C., Otuyalo A.I., Edozie P.K. (2024). Mechanism of the Warburg effect and its role in breast cancer immunotherapy. Discov. Med..

[8] Zhi S., Chen C., Huang H., Zhang Z., Zeng F., Zhang S. (2024). Hypoxia-inducible factor in breast cancer: Role and target for breast cancer treatment. Front. Immunol..

[9] Chetta P., Sriram R., Zadra G. (2023). Lactate as Key Metabolite in Prostate Cancer Progression: What Are the Clinical Implications?. Cancers.

[10] Cakici C., Daylan B., Unluer R.S., Emekli-Alturfan E., Ayla S., Gozel H.E., Yigit P., Dokgoz E.Y., Yigitbasi T. (2024). LDH-A Inhibitor as a Remedy to Potentiate the Anticancer Effect of Docetaxel in Prostate Cancer. J. Cancer.

[11] Almaimani R.A., Aslam A., Ahmad J., El-Readi M.Z., El-Boshy M.E., Abdelghany A.H., Idris S., Alhadrami M., Althubiti M., Almasmoum H.A. (2022). In Vivo and In Vitro Enhanced Tumoricidal Effects of Metformin, Active Vitamin D(3), and 5-Fluorouracil Triple Therapy against Colon Cancer by Modulating the PI3K/Akt/PTEN/mTOR Network. Cancers.

[12] Farrash W.F., Aslam A., Almaimani R., Minshawi F., Almasmoum H., Alsaegh A., Iqbal M.S., Tabassum A., Elzubier M.E., El-Readi M.Z. (2023). Metformin and thymoquinone co-treatment enhance 5-fluorouracil cytotoxicity by suppressing the PI3K/mTOR/HIF1α pathway and increasing oxidative stress in colon cancer cells. Biofactors.

[13] Idris S., Refaat B., Almaimani R.A., Ahmed H.G., Ahmad J., Alhadrami M., El-Readi M.Z., Elzubier M.E., Alaufi H.A., Al-Amin B. (2022). Enhanced in vitro tumoricidal effects of 5-Fluorouracil, thymoquinone, and active vitamin D3 triple therapy against colon cancer cells by attenuating the PI3K/AKT/mTOR pathway. Life Sci..

[14] Boström P., Wu J., Jedrychowski M.P., Korde A., Ye L., Lo J.C., Rasbach K.A., Boström E.A., Choi J.H., Long J.Z. (2012). A PGC1-α-dependent myokine that drives brown-fat-like development of white fat and thermogenesis. Nature.

[15] Brown J.C., Sarwer D.B., Troxel A.B., Sturgeon K., DeMichele A.M., Denlinger C.S., Schmitz K.H. (2021). A randomized trial of exercise and diet on body composition in survivors of breast cancer with overweight or obesity. Breast Cancer Res. Treat..

[16] Ciałowicz M., Woźniewski M., Murawska-Ciałowicz E., Dzięgiel P. (2025). The Influence of Irisin on Selected Organs-The Liver, Kidneys, and Lungs: The Role of Physical Exercise. Cells.

[17] Kurdiova T., Balaz M., Vician M., Maderova D., Vlcek M., Valkovic L., Srbecky M., Imrich R., Kyselovicova O., Belan V. (2014). Effects of obesity, diabetes and exercise on Fndc5 gene expression and irisin release in human skeletal muscle and adipose tissue: In vivo and in vitro studies. J. Physiol..

[18] Moreno-Navarrete J.M., Ortega F., Serrano M., Guerra E., Pardo G., Tinahones F., Ricart W., Fernández-Real J.M. (2013). Irisin is expressed and produced by human muscle and adipose tissue in association with obesity and insulin resistance. J. Clin. Endocrinol. Metab..

[19] Khalili-Tanha G., Shoari A., Nazari E. (2025). The role of Irisin in modulating hypoxia-related disorders: New insights and implications for cancer therapy. Asp. Mol. Med..

[20] Waseem R., Shamsi A., Mohammad T., Hassan M.I., Kazim S.N., Chaudhary A.A., Rudayni H.A., Al-Zharani M., Ahmad F., Islam A. (2022). FNDC5/Irisin: Physiology and Pathophysiology. Molecules.

[21] Farrash W., Brook M., Crossland H., Phillips B.E., Cegielski J., Wilkinson D.J., Constantin-Teodosiu D., Greenhaff P.L., Smith K., Cleasby M. (2020). Impacts of rat hindlimb Fndc5/irisin overexpression on muscle and adipose tissue metabolism. Am. J. Physiol. Endocrinol. Metab..

[22] Maak S., Norheim F., Drevon C.A., Erickson H.P. (2021). Progress and Challenges in the Biology of FNDC5 and Irisin. Endocr. Rev..

[23] Liu S., Cui F., Ning K., Wang Z., Fu P., Wang D., Xu H. (2022). Role of irisin in physiology and pathology. Front. Endocrinol..

[24] Zhang D., Zhang P., Li L., Tang N., Huang F., Kong X., Tan X., Shi G. (2019). Irisin functions to inhibit malignant growth of human pancreatic cancer cells via downregulation of the PI3K/AKT signaling pathway. Onco Targets Ther..

[25] Liu J., Huang Y., Liu Y., Chen Y. (2019). Irisin Enhances Doxorubicin-Induced Cell Apoptosis in Pancreatic Cancer by Inhibiting the PI3K/AKT/NF-κB Pathway. Med. Sci. Monit..

[26] Zhu T., Zhang W., Zhang Y., Lu E., Liu H., Liu X., Yin S., Zhang P. (2022). Irisin/FNDC5 inhibits the epithelial-mesenchymal transition of epithelial ovarian cancer cells via the PI3K/Akt pathway. Arch. Gynecol. Obstet..

[27] Pedersen B.K., Febbraio M.A. (2012). Muscles, exercise and obesity: Skeletal muscle as a secretory organ. Nat. Rev. Endocrinol..

[28] Kim J.S., Taaffe D.R., Galvão D.A., Clay T.D., Redfern A.D., Hart N.H., Gray E.S., Ryan C.J., Kenfield S.A., Saad F. (2023). Acute effect of high-intensity interval aerobic exercise on serum myokine levels and resulting tumour-suppressive effect in trained patients with advanced prostate cancer. Prostate Cancer Prostatic Dis..

[29] Schumacher M.A., Chinnam N., Ohashi T., Shah R.S., Erickson H.P. (2013). The structure of irisin reveals a novel intersubunit β-sheet fibronectin type III (FNIII) dimer: Implications for receptor activation. J. Biol. Chem..

[30] Nie Y., Dai B., Guo X., Liu D. (2020). Cleavage of FNDC5 and insights into its maturation process. Mol. Cell Endocrinol..

[31] Korta P., Pocheć E., Mazur-Biały A. (2019). Irisin as a Multifunctional Protein: Implications for Health and Certain Diseases. Medicina.

[32] Maalouf G.E., El Khoury D. (2019). Exercise-Induced Irisin, the Fat Browning Myokine, as a Potential Anticancer Agent. J. Obes..

[33] Sato S. (2024). Adipo-oncology: Adipocyte-derived factors govern engraftment, survival, and progression of metastatic cancers. Cell Commun. Signal.

[34] Yang Y., Ma X., Li Y., Jin L., Zhou X. (2024). The evolving tumor-associated adipose tissue microenvironment in breast cancer: From cancer initiation to metastatic outgrowth. Clin. Transl. Oncol..

[35] Liermann-Wooldrik K.T., Kosmacek E.A., Oberley-Deegan R.E. (2024). Adipose Tissues Have Been Overlooked as Players in Prostate Cancer Progression. Int. J. Mol. Sci..

[36] Zhang J., Lu E., Deng L., Zhu Y., Lu X., Li X., Li F., Yan Y., Han J.Y., Li Y. (2024). Immunological roles for resistin and related adipokines in obesity-associated tumors. Int. Immunopharmacol..

[37] Bocian-Jastrzębska A., Malczewska-Herman A., Kos-Kudła B. (2023). Role of Leptin and Adiponectin in Carcinogenesis. Cancers.

[38] Nehme R., Diab-Assaf M., Decombat C., Delort L., Caldefie-Chezet F. (2022). Targeting Adiponectin in Breast Cancer. Biomedicines.

[39] Hu X., Hu C., Zhang C., Zhang M., Long S., Cao Z. (2019). Role of Adiponectin in prostate cancer. Int. Braz. J. Urol..

[40] Apostolaki D., Katsibardi K., Efthymiou V., Stefanaki C., Mantzou A., Papadodima S., Chrousos G.P., Kattamis A., Bacopoulou F. (2025). Irisin Concentrations in Children and Adolescent Cancer Survivors and Their Relation to Metabolic, Bone, and Reproductive Profile: A Pilot Case–Control Study. J. Clin. Med..

[41] Tuğral A., Arıbaş Z., Kaya Uçar G., Arslan F.D., Bakar Y., Karakoyun I., Akyol M. (2025). The effect of supervised aerobic exercise on adipokine and myokine biomarkers in patients with cancer during systemic chemotherapy: A single-blinded prospective controlled trial. Support. Care Cancer.

[42] Gannon N.P., Vaughan R.A., Garcia-Smith R., Bisoffi M., Trujillo K.A. (2015). Effects of the exercise-inducible myokine irisin on malignant and non-malignant breast epithelial cell behavior in vitro. Int. J. Cancer.

[43] Tekin S., Erden Y., Sandal S., Yilmaz B. (2015). Is Irisin an Anticarcinogenic Peptide?. Med. Sci. Int. Med. J..

[44] Alshanqiti K.H., Alomar S.F., Alzoman N., Almomen A. (2023). Irisin Induces Apoptosis in Metastatic Prostate Cancer Cells and Inhibits Tumor Growth In Vivo. Cancers.

[45] Saeedi Sadr A., Ehteram H., Seyed Hosseini E., Alizadeh Zarei M., Hassani Bafrani H., Haddad Kashani H. (2022). The Effect of Irisin on Proliferation, Apoptosis, and Expression of Metastasis Markers in Prostate Cancer Cell Lines. Oncol. Ther..

[46] Yang B.C., Leung P.S. (2020). Irisin Is a Positive Regulator for Ferroptosis in Pancreatic Cancer. Mol. Ther. Oncolytics.

[47] Liu J., Song N., Huang Y., Chen Y. (2018). Irisin inhibits pancreatic cancer cell growth via the AMPK-mTOR pathway. Sci. Rep..

[48] Alizadeh Zarei M., Seyed Hosseini E., Haddad Kashani H., Ahmad E., Nikzad H. (2023). Effects of the exercise-inducible myokine irisin on proliferation and malignant properties of ovarian cancer cells through the HIF-1 α signaling pathway. Sci. Rep..

[49] Huang C.W., Chang Y.H., Lee H.H., Wu J.Y., Huang J.X., Chung Y.H., Hsu S.T., Chow L.P., Wei K.C., Huang F.T. (2020). Irisin, an exercise myokine, potently suppresses tumor proliferation, invasion, and growth in glioma. FASEB J..

[50] Kong G., Jiang Y., Sun X., Cao Z., Zhang G., Zhao Z., Zhao Y., Yu Q., Cheng G. (2017). Irisin reverses the IL-6 induced epithelial-mesenchymal transition in osteosarcoma cell migration and invasion through the STAT3/Snail signaling pathway. Oncol. Rep..

[51] Cheng G., Xu D., Chu K., Cao Z., Sun X., Yang Y. (2020). The Effects of MiR-214-3p and Irisin/FNDC5 on the Biological Behavior of Osteosarcoma Cells. Cancer Biother. Radiopharm..

[52] Shao L., Li H., Chen J., Song H., Zhang Y., Wu F., Wang W., Zhang W., Wang F., Li H. (2017). Irisin suppresses the migration, proliferation, and invasion of lung cancer cells via inhibition of epithelial-to-mesenchymal transition. Biochem. Biophys. Res. Commun..

[53] Fan G.H., Zhu T.Y., Huang J. (2020). FNDC5 promotes paclitaxel sensitivity of non-small cell lung cancers via inhibiting MDR1. Cell. Signal..

[54] Moon H.S., Mantzoros C.S. (2014). Regulation of cell proliferation and malignant potential by irisin in endometrial, colon, thyroid and esophageal cancer cell lines. Metabolism.

[55] Shi G., Tang N., Qiu J., Zhang D., Huang F., Cheng Y., Ding K., Li W., Zhang P., Tan X. (2017). Irisin stimulates cell proliferation and invasion by targeting the PI3K/AKT pathway in human hepatocellular carcinoma. Biochem. Biophys. Res. Commun..

[56] Yang J., Weinberg R.A. (2008). Epithelial-mesenchymal transition: At the crossroads of development and tumor metastasis. Dev. Cell.

[57] Loh C.Y., Chai J.Y., Tang T.F., Wong W.F., Sethi G., Shanmugam M.K., Chong P.P., Looi C.Y. (2019). The E-Cadherin and N-Cadherin Switch in Epithelial-to-Mesenchymal Transition: Signaling, Therapeutic Implications, and Challenges. Cells.

[58] Provatopoulou X., Georgiou G.P., Kalogera E., Kalles V., Matiatou M.A., Papapanagiotou I., Sagkriotis A., Zografos G.C., Gounaris A. (2015). Serum irisin levels are lower in patients with breast cancer: Association with disease diagnosis and tumor characteristics. BMC Cancer.

[59] Zhang Z.P., Zhang X.F., Li H., Liu T.J., Zhao Q.P., Huang L.H., Cao Z.J., He L.M., Hao D.J. (2018). Serum irisin associates with breast cancer to spinal metastasis. Medicine.

[60] Kuloglu T., Celik O., Aydin S., Hanifi Ozercan I., Acet M., Aydin Y., Artas G., Turk A., Yardim M., Ozan G. (2016). Irisin immunostaining characteristics of breast and ovarian cancer cells. Cell. Mol. Biol..

[61] Zhu H., Liu M., Zhang N., Pan H., Lin G., Li N., Wang L., Yang H., Yan K., Gong F. (2018). Serum and Adipose Tissue mRNA Levels of ATF3 and FNDC5/Irisin in Colorectal Cancer Patients With or Without Obesity. Front. Physiol..

[62] Wozniak S., Nowinska K., Chabowski M., Dziegiel P. (2022). Significance of Irisin (FNDC5) Expression in Colorectal Cancer. In Vivo.

[63] Esawy M.M., Abdel-Samd K.M. (2020). The diagnostic and prognostic roles of serum irisin in bladder cancer. Curr. Probl. Cancer.

[64] Aslan R., Alp H.H., Eryılmaz R., Huyut Z., Sevim M., Araz Ş., Ertas K., Taken K. (2020). Can the Irisin be a Biomarker for Prostate Cancer? A Case Control Study. Asian Pac. J. Cancer Prev..

[65] Gaggini M., Cabiati M., Del Turco S., Navarra T., De Simone P., Filipponi F., Del Ry S., Gastaldelli A., Basta G. (2017). Increased FNDC5/Irisin expression in human hepatocellular carcinoma. Peptides.

[66] Zhang J., Ke M., Ren Y., Bi J., Du Z., Zhang M., Wang Y., Zhang L., Wu Z., Lv Y. (2019). Serum Irisin Predicts Posthepatectomy Complications in Patients with Hepatocellular Carcinoma. Dis. Markers.

[67] Pazgan-Simon M., Zuwała-Jagiełło J., Kukla M., Grzebyk E., Simon K. (2020). Serum concentrations of selected adipokines in virus-related liver cirrhosis and hepatocellular carcinoma. Clin. Exp. Hepatol..

[68] Shahidi S., Hejazi J., Moghimi M., Borji S., Zabihian S., Fathi M. (2020). Circulating Irisin Levels and Redox Status Markers in Patients with Gastric Cancer: A Case-Control Study. Asian Pac. J. Cancer Prev..

[69] Aydin S., Kuloglu T., Ozercan M.R., Albayrak S., Aydin S., Bakal U., Yilmaz M., Kalayci M., Yardim M., Sarac M. (2016). Irisin immunohistochemistry in gastrointestinal system cancers. Biotech. Histochem..

[70] Altay D.U., Keha E.E., Karagüzel E., Menteşe A., Yaman S.O., Alver A. (2018). The Diagnostic Value of FNDC5/Irisin in Renal Cell Cancer. Int. Braz. J. Urol..

[71] Kuloğlu T., Artaş G., Yardim M., Sahin I., Aydin Y., Beyoğlu N., Özercan I.H., Yalcin M.H., Ugur K., Aydin S. (2019). Immunostaining characteristics of irisin in benign and malignant renal cancers. Biotech. Histochem..

[72] Ugur K., Aydin S., Kuloglu T., Artas G., Kocdor M.A., Sahin İ., Yardim M., Ozercan İ.H. (2019). Comparison of irisin hormone expression between thyroid cancer tissues and oncocytic variant cells. Cancer Manag. Res..

[73] Nowinska K., Jablonska K., Pawelczyk K., Piotrowska A., Partynska A., Gomulkiewicz A., Ciesielska U., Katnik E., Grzegrzolka J., Glatzel-Plucinska N. (2019). Expression of Irisin/FNDC5 in Cancer Cells and Stromal Fibroblasts of Non-small Cell Lung Cancer. Cancers.

[74] Pinkowska A., Podhorska-Okołów M., Dzięgiel P., Nowińska K. (2021). The Role of Irisin in Cancer Disease. Cells.

[75] Albrecht E., Norheim F., Thiede B., Holen T., Ohashi T., Schering L., Lee S., Brenmoehl J., Thomas S., Drevon C.A. (2015). Irisin—A myth rather than an exercise-inducible myokine. Sci. Rep..

[76] Crujeiras A.B., Pardo M., Casanueva F.F. (2015). Irisin: ‘fat’ or artefact. Clin. Endocrinol..

[77] Albrecht E., Schering L., Buck F., Vlach K., Schober H.C., Drevon C.A., Maak S. (2020). Irisin: Still chasing shadows. Mol. Metab..

[78] Mebratie D.Y., Dagnaw G.G. (2024). Review of immunohistochemistry techniques: Applications, current status, and future perspectives. Semin. Diagn. Pathol..

